# Experience of Using Neural Networks to Assess Age-Related Changes in Some Structures of the Skull and Cervical Vertebrae Based on CT Scans (Pilot Project)

**DOI:** 10.17691/stm2024.16.2.03

**Published:** 2024-04-27

**Authors:** G.V. Zolotenkova, D.K. Valetov, M.P. Poletaeva, Yu.V. Vassilevski

**Affiliations:** MD, DSc, Professor, Department of Forensic Medicine; First Moscow State Medical University (Sechenov University), 8/2 Malaya Trubetskaya St., Moscow, 119991, Russia; Tutor, Department of Higher Mathematics, Mechanics, and Mathematical Modeling, Institute of Computer Science and Mathematical Modeling of the Biomedicine Science and Technology Park; First Moscow State Medical University (Sechenov University), 8/2 Malaya Trubetskaya St., Moscow, 119991, Russia; MD, PhD, Associate Professor, Department of Forensic Medicine; First Moscow State Medical University (Sechenov University), 8/2 Malaya Trubetskaya St., Moscow, 119991, Russia; DSc, Professor, Corresponding Member of the Russian Academy of Sciences, Head of the Department of Higher Mathematics, Mechanics, and Mathematical Modeling, Institute of Computer Science and Mathematical Modeling of the Biomedicine Science and Technology Park; First Moscow State Medical University (Sechenov University), 8/2 Malaya Trubetskaya St., Moscow, 119991, Russia

**Keywords:** age estimation, CT scans, atlantoaxial joint, vertebrae, deep learning, artificial neural networks

## Abstract

**The aim of the investigation** is to study the possibility of using artificial intelligence technologies for age prediction based on CT studies of some structures of the skull and cervical vertebrae.

**Material and Methods:**

The study included 223 tomograms of the head and neck in sagittal projection from patients without any pathology of the studied structures. Morphometric analysis was carried out using PjaPro and Gradient programs, statistical analysis was performed by SPSS Statistics software. A fully convolutional EfficientNet-B2 neural network was used, which was trained in two stages: selection of the area of interest and solution of regression tasks.

**Results:**

Morphometric assessment and subsequent statistical analysis of the selected group of features have shown presence of the strongest correlation with age in the indicator characterizing the involution of the median atlantoaxial joint. A deep learning method using the convolutional network, which automatically selects the desired area in the image (the area of the vertebral junction), classifies the sample, and makes an assumption about the age of the unknown individual with an accuracy of 7.5 to 10.5 years has been tested.

**Conclusion:**

As a result of the study, a positive experience has been obtained indicating the possibility of using convolutional neural networks to determine the age of the unknown person, which expands the evidence base and provides new opportunities for determining group-wide personality traits in forensic medicine.

## Introduction

In the current realities, forensic medicine is highly demanded for age estimation [1, 2]. It may be accounted for, in particular, by the growth of migration activity and, as a consequence, the increase of people on the territory of an alien country without any identity documents [3, 4].

There are a lot of methods of age estimation based on the qualitative or quantitative analysis of morphological parameters of bone structures and their radiological images [2, 5]. The radiological techniques have some drawbacks, the main of being low accuracy of the results and subjectivity in the assessment of the obtained data. Besides, they are often sufficiently labor-consuming. Radiological methods of medical imaging have a number of advantages over ordinary radiographs. For example, CT gives the possibility to study precisely and in greater detail not only an organ but separate structures, their organization, and the bone tissue itself owing to the acquisition of high-resolution images of various layers without overlaying.

Presently, there is a demand in developing new methods, which are fast and convenient in the routine application and may help identify a biological age using up-to-day computer programs able to provide reproducibility of the method itself and reliability of expert conclusions [6–8]. A fully automated, impartial, and noninvasive method for accurate determination of human age must be the final result of this work.

The technologies of machine learning have proved to be a powerful and effective tool of working with medical images and made it possible to optimize many routine diagnostic processes. For example, traditional algorithms, based on manual programming of functions, were successfully replaced owing to the implementation of a computer vision [9–12]. In medical practice, deep neural networks are employed to detect patterns of interstitial lung diseases on the CT scans of the thoracic organs [13, 14]; for segmentation of the human vascular eye network on the photographs of the ocular bottom [15]; prediction of coronary microvascular obstruction phenomenon developing in the process of percutaneous coronary interventions [16]; and also for estimation of age by hand [17] and teeth [8] rentgenograms. An important advantage of the deep neural networks is the ability to obtain high-level hierarchical image representation [18]. In addition to the tasks of segmentation and localization, many methods based on deep learning are well suited for the tasks of regression and classification in medical imaging, which also allowed for their application for age assessment in forensic medicine [2, 5].

Machine learning methods are considered promising for reducing the dependence of the results on the user with simultaneous acceleration of the process and standardization of measurements. Recent achievements in the field of artificial intelligence allowed for automatization of the working processes and obtaining new results in medicine [18, 19]. Artificial neural networks (ANN), serving as function selectors, may be trained to extract information pertaining to a specific task [20]. All the above mentioned shows that the development and implementation of neural networks for estimating the age changes are of great importance.

**The aim of the investigation** is to study the possibility of using artificial intelligence technologies for age prediction based on CT examinations of some structures of the skull and cervical vertebrae.

## Materials and Methods

### Materials

CT skull scanograms of the patients examined in the Diagnostic Radiology Units of University Clinical Hospital No.1 of the First Moscow State Medical University (Sechenov University, Russia) in the period from 2018 to 2022. All investigations were carried out on the Canon Aguillion One computed tomography system (Toshiba, Japan) with 320 detector rows and 0.5 mm slice thickness. The obtained data were exported to the DICOM image format and then the Merge Healthcare– eFilm Workstation 4.2 program was used to export the sagittal skull projections in bone mode to the BMP format for further investigations.

### Sample description

**Figure 1.**
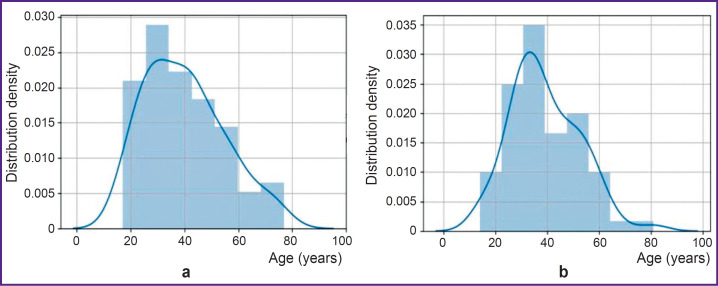
Distribution of samples according to gender and age: (a) women, (b) men

The study included 223 sagittal projections of anonymized tomograms of the head and neck of patients at the age from 17 to 79 years without any pathology of the structures of the internal cranial base and craniovertebral region, i.e. frontal, sphenoid sinuses, and C1, C2 cervical vertebrae. The person’s age in years was calculated on the basis of the date of birth and the date of image acquisition. The ratio of men and women in the sample was 54 and 46%, respectively, filtration was not applied. Age distributions between men and women were almost equal. The data were shifted towards the younger samples although the average age was 39 years, which statistically classifies the samples as “adults”. The age groups over 60 years have a significantly smaller size. The detailed sample distribution according to gender and age is presented in Figure 1.

***Morphometric analysis*** of the CT images was performed using the PjaPro program designed for the automatic selection of the “spots” with preservation of coordinates and parameters in the file (assessment of geometrical characteristics of images of frontal and sphenoid sinuses, features 1–17 in the Table), and the Gradient program for the analysis of diffused color or grayscale images (assessment of non-uniformity of bone tissue image in the region of the Blumenbach’s clivus and clinoid plate, features 18–25 in the Table) [21]. Based on the obtained initial values, we calculated in PjaPro the value of an average sinus dimension, the ratio of frontal sinus areas to the area of the sphenoid sinus in relative units (pixels), and two new dimensionless values characterizing the degree of sinus image elongation, i.e. mean and maximum values of sinus elongation. This methodology of measuring the examined features is presented in the previous work [22].

**Table:** A list of parameters for measurements in Gradient and PjaPro programs with the coefficient of correlation between the examined parameters and age

Feature No.	Parameter	Correlation coefficient (with age)	p
1	Number of dots in the frontal sinus	0.199	0.076
2	Mean squared distance of dots in the frontal sinus from the center of the sinus image	0.136	0.229
3	Maximum distance between the dots in frontal sinus	0.104	0.357
4	Direction, along which frontal sinus image is more elongated	–0.233	0.037
5	Number of dots in sphenoid sinus	–0.027	0.809
6	Mean squared distance between the dots in sphenoid sinus	0.026	0.82
7	Maximum distance between the dots in sphenoid sinus	–0.015	0.893
8	Direction, along which sphenoid sinus image is more elongated	0.009	0.935
9	Horizontal distance between the centers of frontal and sphenoid sinuses	–0.197	0.08
10	Vertical distance between the centers of frontal and sphenoid sinuses (delta_Y)	0.312	0.005
11	Average size of frontal sinuses	0.232	0.038
12	Average value of frontal sinus elongation	–0.066	0.563
13	Maximum value of frontal sinus elongation	–0.066	0.559
14	Average size of sphenoid sinus	0.014	0.903
15	Average value of sphenoid sinus elongation	0.081	0.476
16	Maximum value of sphenoid sinus elongation	–0.072	0.524
17	Ratio of frontal/sphenoid sinus areas	0.23	0.04
18	Average value of gray brightness of dots of bone substance structure of Blumenbach’s clivus and clinoid plate	–0.455	<0.001
19	Dispersion of dot brightness of the bone substance structure of Blumenbach’s clivus and clinoid plate	–0.741	<0.001
20	Mean squared deviation of dot brightness of the bone substance structure of Blumenbach’s clivus and clinoid plate	–0.761	<0.001
21	Coefficient of variation of dot brightness of the bone substance structure of Blumenbach’s clivus and clinoid plate	–0.581	<0.001
22	Third central moment of dot brightness of the bone substance structure of Blumenbach’s clivus and clinoid plate	0.072	0.523
23	Coefficient of asymmetry of dot brightness of the bone substance structure of Blumenbach’s clivus and clinoid plate	0.385	<0.001
24	Fourth central moment of dot brightness of the bone substance structure of Blumenbach’s clivus and clinoid plate	–0.672	<0.001
25	Coefficient of excess of dot brightness of the bone substance structure of Blumenbach’s clivus and clinoid plate	0.658	<0.001
26	Involution of Cruveilhier joint	0.885	<0001

The next step of the study consisted in the analysis of the vertebra ratio in the atlantoaxial Cruveilhier joint by means of visual morphometric method of studying CT images. The sample was additionally splitted into the age groups with a 10-year interval for convenient evaluation of the involutive changes of the joint.

A common list of the examined features (parameters) composed on the basis of studying specialized literature on age morphology (see the Table) has been formed.

### Neuronal networks

3D images were acquired from the DICOM unprocessed files by converting them into the .nii files. Then, the images were loaded to Python in the form of NumPy arrays with the help of SimpleITK library. The resulting 3D images had a resolution of 512×512×Z, where Z varied in the range from 197 to 631. A fully convolutional EfficientNet-B2 neural network [20] was used for selecting the area of interest and a neural network consisting mainly of the layers of a custom 3D architecture was employed for solving the task of age regression. To assess the accuracy and effectiveness of the classification task being solved by the neural networks, we used data visualization method known as a confusion or error matrix [18, 19], while to assess the quality of the regression task solution we applied a dispersion diagram for training and test data.

### Statistical analysis

The results of morphometric study were statistically processed using the SPSS Statistics 21 program. A descriptive statistics method was applied with computation of the mean arithmetic value of statistical error; mean square deviation, median, minimum and maximum feature values. Normality of distribution was calculated using the Kolmogorov– Smirnov test. Some indicators had a strong noncompact and asymmetric distribution (features 24 and 25). For the indicators with a noncompact and asymmetric distribution, nonparametric statistical methods were applied, therefore, not only the arithmetic mean but also median with quartiles were counted. To check the mutual effect of the factors, we used the correlation analysis method with calculation of the Pearson coefficient of correlation (r). Statistical significance of differences was determined using the Mann–Whitney U-test considering the differences statistically significant at p<0.05.

## Results

**Figure 2.**
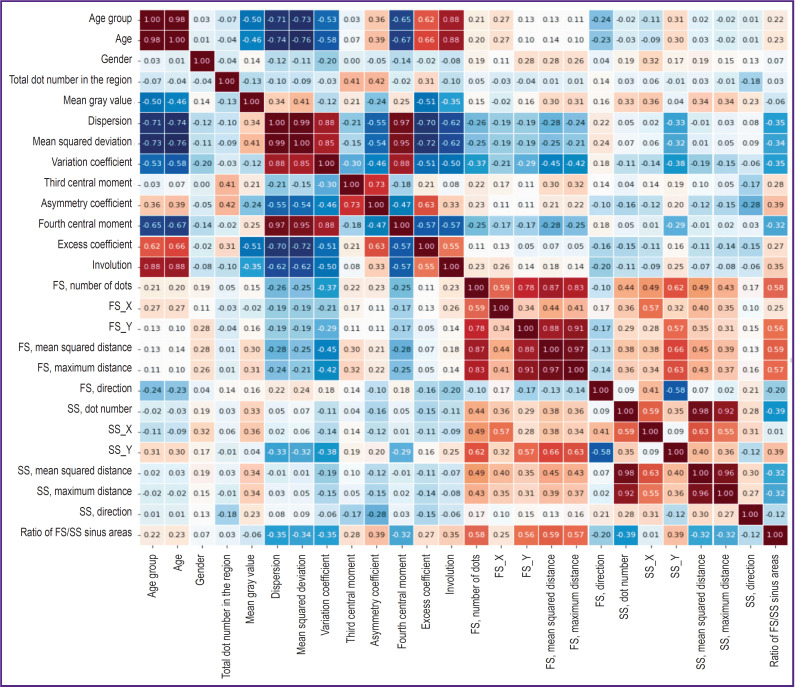
Heatmap of indicators of age changes of the examined cranial structures FS — frontal sinus, SS — sphenoid sinus

The morphometric assessment of age changes in frontal and sphenoid sinuses consisted in determining the geometry of sinuses and their dimensional characteristics using the PjaPro program [16, 18]. In the process of our investigation, 17 metric parameters of the frontal and sphenoid sinuses have been analyzed (features 1–17 in the Table). Additionally to the metric dimensions of the frontal and sphenoid sinuses, the coefficients of their ratios and differences in location of conditional centers of sinuses along the vertical axis were calculated. The statistical analysis has established insignificant correlation between the examined indicators and age. Consequently, these parameters (see the Table, Figure 2) cannot be further used in our work.

### Studying age changes in the region of clinoid plate and Blumenbach’s clivus

The intensity and prevalence of bone substance rarefaction on CT scans have been analyzed in order to evaluate age changes in the area of the clinoid plate and Blumenbach’s clivus. The Gradient program was used to assess non-uniformity of bone tissue color of these anatomic structures. The degrees of brightness were determined for each dot of the bone tissue in the range of 0 (black) to 255 (brightly white), and the obtained set of the dot brightness was evaluated as a number of observations of a random variable. Nine parameters (features 18–25 in the Table) were chosen for further investigations. A heatmap, presented in Figure 2, was built for visual analysis of feature relationships.

The correlation analysis has established the following: a mean value of gray brightness for the dots for the bone substance structures of Blumenbach’s clivus and clinoid plate (feature 18) decreases with age (becomes darker on the heatmap); dispersion (feature 19) and the mean squared deviation of brightness (feature 20) become more uniform with age; the variation coefficient (feature 21) and the third central moment of brightness (feature 22) do not have statistically significant correlations with age; the fourth central moment (feature 24) and the excess coefficient (feature 25) are distributed less compactly depending on the number of years lived. The strongest correlation with age was found in the indicator “the mean squared deviation of brightness for the bone substance structures of Blumenbach’s clivus and clinoid plate” (feature 20). Based on the exploration of the brightness dot distribution, the interrelations of age with the specifics of bone tissue images were shown. The characteristics reflecting in whole the processes of changes in the density of the bone tissue, its rarefaction and non-uniformity, which are progressing with age, have been captured.

### Morphological study of transformation (involution) of the median atlantoaxial joint (Cruveilhier joint)

Morphological changes in C1 and C2 cervical vertebrae were assess in dynamics, the interrelation between the vertebrae was also analyzed during morphometric study of transformation (involution) of the median atlantoaxial joint (Cruveilhier joint). The dynamics of the age transformation of this joint is described in detail in our work [22].

### Morphological study of transformation (involution) of the median atlantoaxial joint (Cruveilhier joint)

Morphological changes in C1 and C2 cervical vertebrae were assess in dynamics, the interrelation between the vertebrae was also analyzed during morphometric study of transformation (involution) of the median atlantoaxial joint (Cruveilhier joint). The dynamics of the age transformation of this joint is described in detail in our work [22].

**Figure 3.**
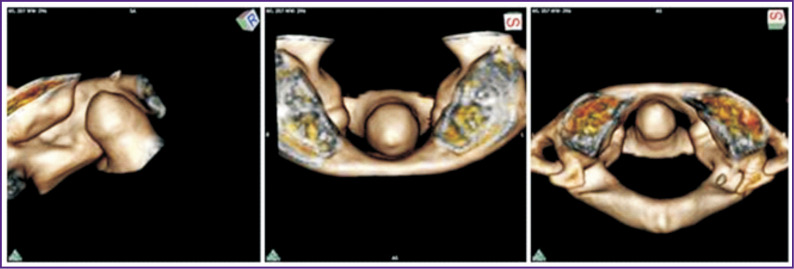
3D reconstruction of CT image of median atlantoaxial joint for an 18-year old man

0 points (age range — 14–20 years): surfaces of the anterior atlas arch and the dens axis are regular and smooth, contours are somewhat angular. The distance between the anterior surface of the dens axis and internal surface of the atlas arch in the upper third is about 3 mm on average. The dens apex is, as a rule, higher than a visible region of the atlas arch. The anterior atlas arch is positioned at an acute angle, which is open upwardly relative to the anterior surface of the dens axis (Figure 3).

1 point (age range — 21–30 years): the surfaces of the anterior arch of atlas and dens axis are regular. The dens apex is, as a rule, higher at the level of the visible upper region of the atlas arch but may be slightly higher. The anterior atlas arch is located at an acute angle, which is open upwardly relative to the anterior surface of the dens axis. The distance between the anterior surface of the dens axis and internal surface of the atlas arch in the upper third is about 2–3 mm on average. A tendency of the anterior atlas arch to the parallel location relative to the anterior surface of the dens axis (C2) is noted.

2 points (age range — 31–39 years): the surfaces of the anterior atlas arch and dens axis are regular, but some roughness is observed by the end of this age interval due to the bone exostoses appearing for the first time and located in the region of the upper margin of the atlas arch. The distance between the anterior surface of the dens axis and internal surface of the atlas arch in the upper third is on average about 2–3 mm. Contours of the anterior atlas arch acquire a linear-arcuate shape.

3 points (age range — 40–49 years): in the majority of cases, the anterior atlas arch is located in parallel with anterior articular surface of the dens axis. The distance between the anterior surface of the dens axis and internal surface of the atlas arch in the upper third is about 1–2 mm on average. The surfaces are rough due to bone exostoses. Bone exostoses are determined in the region of the superior margin of the atlas arch. In this age interval, formation of exostoses is also noted in the region of the inferior margin of the atlas arch and the apex of the dens axis. By the end of the age period, cases of the arcuate location of the surfaces of the median atlanto-axial joint are noted, but the arch in this case is open posteriorly.

4 points (age range — 50–59 years): the anterior atlas arch is located either parallel rectilinearly to the anterior articular surface of the dens axis or slightly wider in the superior part of the median atlantoaxial joint than in its inferior part. The distance between the anterior surface of the dens axis and internal surface of the atlas arch in the upper third is on average about 1 mm. Bone exostoses in the region of the superior margin, inferior margin of atlas arch, and on the dens apex of the axis vertebra become clearly marked.

5 points (age range — 60–70 years) — the anterior atlas arch is located parallel rectilinearly or parallel arcuately to the anterior articular surface of the dens axis, which is noted along the entire surfaces of the median atlantoaxial joint. The distance between the anterior surface of the dens axis and internal surface of the atlas arch in the upper third is about 1 mm on average. Bone exostoses in the region of the superior margin, inferior margin of the atlas arch, and on the dens apex of the axis vertebra are clearly marked; due to the emergence of exostoses, some angular roughness on the surfaces of the anterior atlas arch and dens axis is formed.

**Figure 4.**
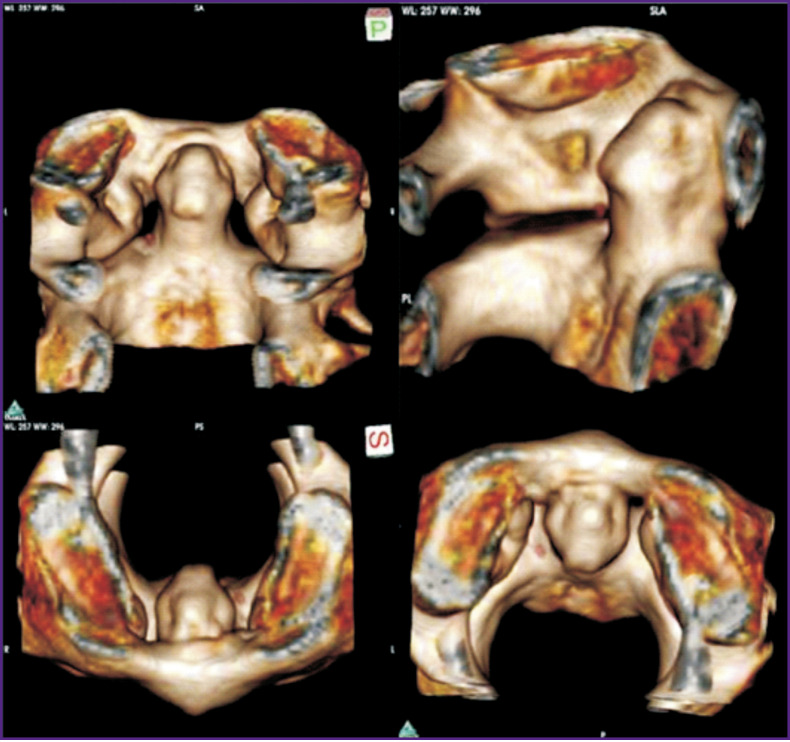
3D reconstruction of a CT image fragment of median atlantoaxial joint for a 69-year old man

6 points (over 71 years): the anterior atlas arch is located parallel to the anterior articular surface of the dens axis. The distance between the anterior surface of the dens axis and internal surface of the atlas arch in the upper third is on average less than 1 mm. Bone exostoses in the region of the superior margin, inferior margin of the atlas arch, and on the dens apex are, as a rule, clearly marked (Figure 4).

All examined images were evaluated according to the presented classification scale. As a result of statistical processing, a strong direct relationship between age and involution value of the median atlantoaxial joint has been detected (feature 26); r=0.89; p<0.001 (see Figure 2).

***Exploration of age-related changes in the region of craniovertebral junction using neural networks*** allowed us to reveal features reflecting most accurately degenerative processes in bone tissues. Among all features of the examined group, the greatest correlation was observed in feature 26, involution of Cruveilhier joint, therefore, further work was focused at this feature and consisted of two stages: selection of the region of interest (showing a general aging tendency) and solution of the regression task.

*The first stage* — selection of the region of interest, which is the vicinity of the atlas and axis vertebrae. Three points were found for each image: the atlas point with the highest position (maximum Z-coordinate), atlas point with the lowest position (minimum Z-coordinate), apex of the dens axis (odontoid process) with the highest position (maximum Z-coordinate). Coordinates for each point, Zmin and Zmax, and for the tip of the dens axis, X0, Y0, Z0, were saved. These values must be determined automatically to generate the region of interest — [X0–64, X0+64]×[Y0–64, Y0+64]× ×[Zmin, Zmax].

**Figure 5.**
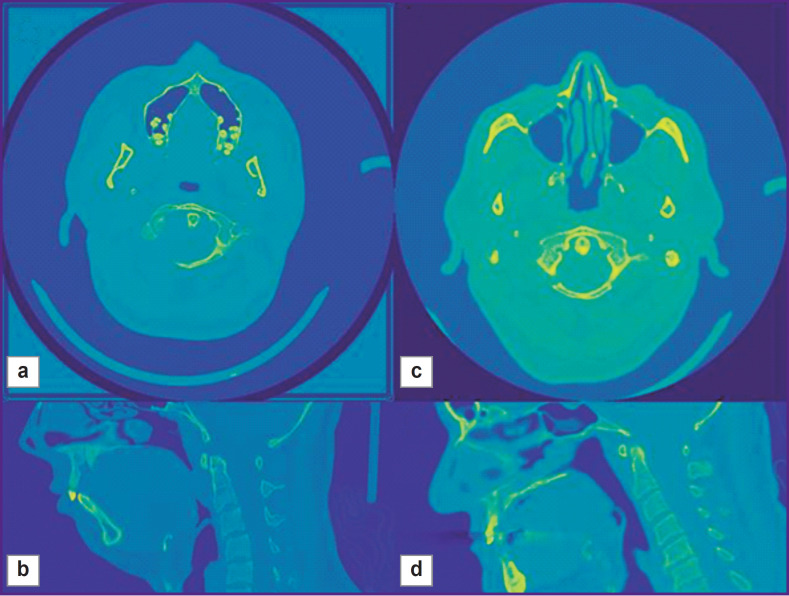
Fragments of CT images used for machine learning in axial and horizontal regions for a 25-year old man (a), (b) and a 57-year old man (c), (d): (a), (c) horizontal projection; (b), (d) sagittal projection

For this purpose, we used a convolutional neural network EfficientNet-B2 [20], which simultaneously classifies 2D slices in the axial, sagittal, and coronal projections (Figure 5) and assigns label 1 (interesting class) if it crosses the region of interest; all other slices receive label 0. A training set of images was supplied with annotations having specified manually the above-mentioned coordinates. The configuration of the neural network training was as follows: the package size was 128 2D images, the size of 2D fragments was changed to 128 pixels in height and width, application of the Adam optimizer with the learning rate of 0.000001 (dimensionless quantity) and cosine regulation of the learning rate with annealing, 10 passes through the training dataset. After learning, the Slicer model is able to extract 3D regions of interest.

**Figure 6.**
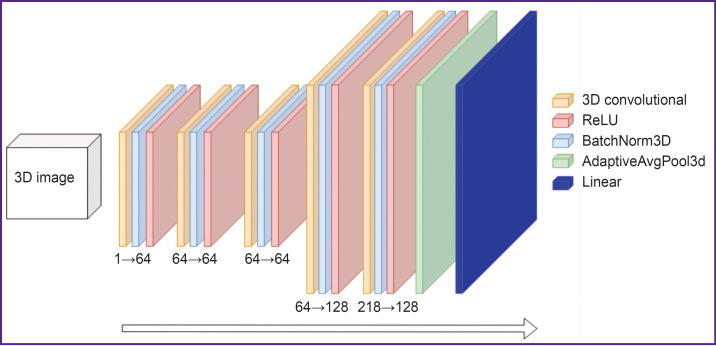
Architecture of the 3D convolutional network Numerals in “a→b” format describe conversion of the channel number through the layers

*The second stage*, solving a regression task for age prediction, is implemented using 3D convolutional neural network, whose structure is demonstrated in Figure 6.

The configuration of the neural network training is as follows: the package size equal to 16 3D images, application of the Adam optimizer with the learning rate of 0.001 (dimensionless quantity) with the regulation of the learning rate based on a cosine function with annealing. All 3D regions were reduced to the 64×64×64 size before their transmission to the neural network; 150 passes through the training dataset.

The initial image set was randomly divided into training and test subsets in the ratio 4:1. During training, usual image augmentations were used (rotation, inversion, and mirroring). Normalization of the target indicator (age) did not cause any significant effect on the final result.

**Figure 7.**
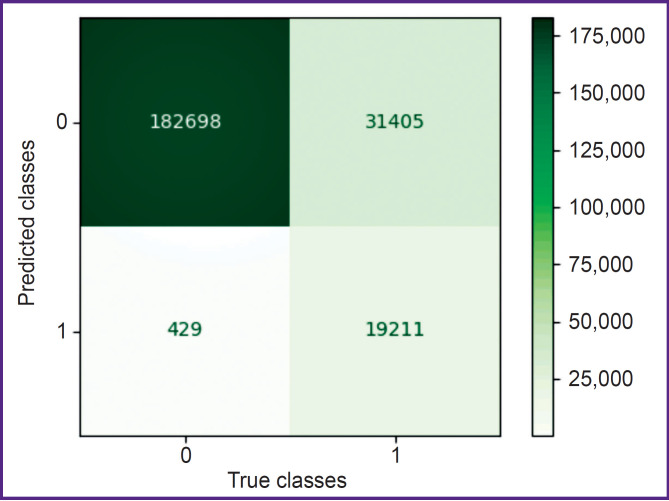
Confusion matrix based on the test data for the neural network

**Figure 8.**
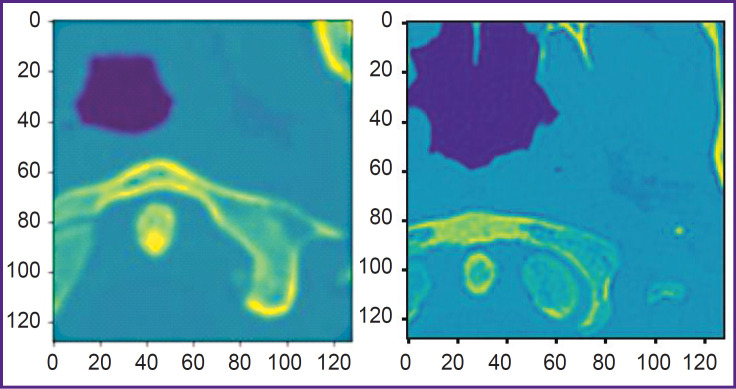
Examples of the regions of interest (Z-shaped notch) Apex of the dens axis is shaped as a circle and is surrounded by the atlas. The atlas does not form a loop, since the notch crosses it at some angle

Using all these training configurations, the following results have been obtained: at the first stage, consisting in the selection of the region of interest, a confusion matrix was generated (Figure 7). The precision of class 0 is close to 1.0; recall for class 1 is equal to 0.98. However, the precision for class 1 is 0.38. This precision is insufficient for the application of all images with label 1. Therefore, the region of interest was selected with the help of a procedure, which ensures the inclusion of the space between the atlas and axis. First, we calculated Zm, an average Z-coordinate of XY slices with label 1; Xm, average X-coordinate of all ZY slices with label 1, and Ym, average Y-coordinate of all ZX slices with label 1. The next step included STD calculation, i.e. standard deviation of Z-coordinates for XY slices with label 1. The region of interest is represented by a field [Xm–64, Xm+64]×[Ym–64, Ym+64]×[Zm–STD, Zm+STD]. Two examples of the extracted regions are presented in Figure 8.

**Figure 9.**
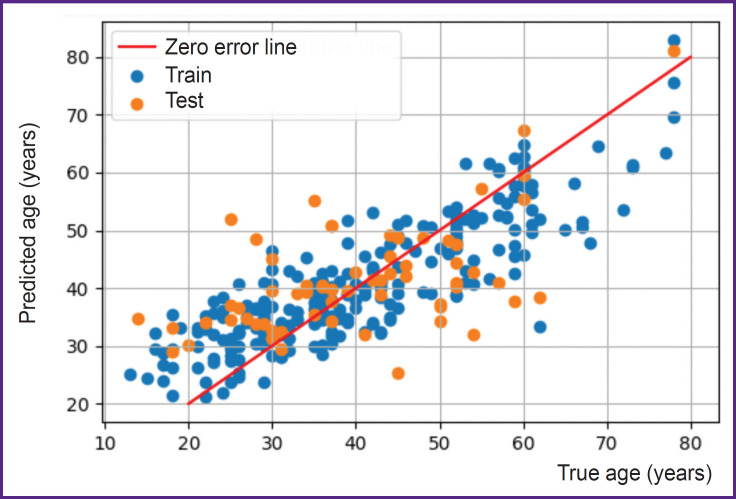
A scatterplot of the neuronet predictions for training and test data

The aim of the second stage was to solve the regression task for age prediction. Figure 9 shows a scatter plot for training and test data. As a result of learning, a mean squared error on the training data was 7.5 years, and on the test data 10.5 years.

## Discussion

According to Dallora et al. [12], the most demanding objects for age identification using machine learning are images of hand and wrist bones. To acquire the images, the majority of the authors choose rentgenography, MRT is encountered rarely, and CT was used only in one study. Machine learning appeared to be the most widely spread intellectual method of evaluation based on the regression analysis. Besides, a small number works are reported to be devoted to the development of the methods for assessing bone age using convolutional neural networks despite the positive results achieved by their application.

A small range of objects and investigation methods, and the prevalence of works from the USA and Western Europe imply receiving limited data from specific population. The majority of publications are devoted to determining the age of children and adolescents, and only a small number to the adult age [12]. This circumstance as well as insufficient information on the possibility of using images of the skull and cervical vertebrae for diagnosing bone age in adults defined our aim and choice of the object for the present investigation.

In the work presented, age changes of various anatomic cranial regions and cervical vertebrae have been analyzed by means of convolutional neural networks. Computer-assisted and automatic solution for estimating biological age based on CT images has been proposed. The results of the present pilot project demonstrate the effectiveness of this innovation approach and the potential of attracting artificial intelligence for solving this kind of tasks. An important advantage of the designed model is the possibility of using it for identifying the age of living individuals and when performing virtopsy. The current complicated geopolitical situation sometimes requires expert investigations to identify a person, which underlines the state and social significance of the present study.

## Conclusion

Age variability of some cranial structures has been studied based on the data of CT images. Among 26 features, a strong correlation with age was established in the indicator characterizing the value of involution of the median atlantoaxial joint. In this connection, the Cruveilhier joint became a region of interest for estimating age changes and chronologic age prediction of the examined persons. As a result of the study, we have obtained positive experience of applying a deep learning system using a convolutional neural network, which automatically selects the region of interest on the CT scan, classifies the specimen, and predicts the age of the unknown individual with the precision of 7.5–10.5 years. The generated and trained model has demonstrated good results, making it possible to develop and improve this method and implement it into practice. In future, it is planned to widen the database and add more information to improve further learning and age prediction accuracy.
